# Using Biomass Gasification Mineral Residue as Catalyst to Produce Light Olefins from CO, CO_2_, and H_2_ Mixtures

**DOI:** 10.1002/cssc.202200436

**Published:** 2022-03-28

**Authors:** Iris C. ten Have, Robin Y. van den Brink, Stéphane C. Marie‐Rose, Florian Meirer, Bert M. Weckhuysen

**Affiliations:** ^1^ Inorganic Chemistry and Catalysis Debye Institute for Nanomaterials Science Utrecht University Universiteitsweg 99 3584 CG Utrecht Netherlands; ^2^ Westbury Innovation Center Enerkem Inc. 551 Chemin des Tuileries J0B 1R0 Westbury, QC Canada

**Keywords:** biomass residue, CO_2_ hydrogenation, Fischer–Tropsch, iron, olefins

## Abstract

Gasification is a process to transform solids, such as agricultural and municipal waste, into gaseous feedstock for making transportation fuels. The so‐called coarse solid residue (CSR) that remains after this conversion process is currently discarded as a process solid residue. In the context of transitioning from a linear to a circular society, the feasibility of using the solid process residue from waste gasification as a solid catalyst for light olefin production from CO, CO_2_, and H_2_ mixtures was investigated. This CSR‐derived catalyst converted biomass‐derived syngas, a H_2_‐poor mixture of CO, CO_2_, H_2_, and N_2_, into methane (57 %) and C_2_–C_4_ olefins (43 %) at 450 °C and 20 bar. The main active ingredient of CSR was Fe, and it was discovered with operando X‐ray diffraction that metallic Fe, present after pre‐reduction in H_2_, transformed into an Fe carbide phase under reaction conditions. The increased formation of Fe carbides correlated with an increase in CO conversion and olefin selectivity. The presence of alkali elements, such as Na and K, in CSR‐derived catalyst increased olefin production as well.

## Graphical abstract

**Waste not, want not**: Repurposing industrial waste as a solid catalyst to produce olefins from CO/CO_2_ represents an elegant possibility towards increased sustainability and materials circularity. The catalyst converted biomass‐derived syngas, a H_2_‐poor mixture of CO, CO_2_, H_2_, and N_2_, into methane (57 %) and C_2_–C_4_ olefins (43 %) at 450 °C and 20 bar.

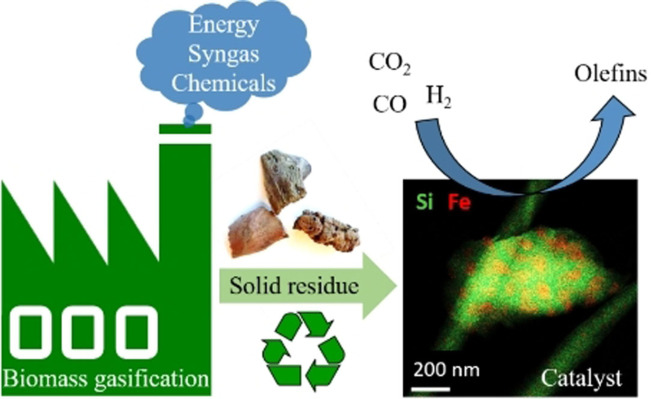

## Introduction

Ceaselessly increasing both global greenhouse gas emissions and energy demand while depleting fossil resources constitutes a major issue for today's society. To solve this, radical changes in awareness, mindset, and behavior of both the consumer and industry are inevitable. Currently, we are living in a mostly linear economy; resources are converted to products and disposed after usage. Efforts are being made to transition to a circular economy, where resources are recycled.^[1]^ It is thus indispensable to explore potential solutions and set off in new directions. On the one hand, we need to find efficient ways to mitigate anthropogenic emissions of greenhouse gases, such as CO_2_, and reverse global warming effects.^[2]^ On the other hand, alternative feedstocks are required to meet the needs of the further increasing energy demand.[^3^, ^4^, ^5^] During the last years, the awareness of proceeding climate change and the urgent need to act grew, which forced the implementation of several climate change mitigation policies.^[2]^ Nevertheless, the global CO_2_ emissions are predicted to continuously increase. To solve this issue, carbon capture and storage (CCS) and carbon capture and utilization (CCU) are promising options. Especially the efficient conversion of captured CO_2_ into value‐added products, including fuels and chemical building blocks, could be a significant breakthrough.[^6^, ^7^] In this context, thermochemical CO_2_ hydrogenation towards value‐added products gained attraction, as the broad range of possible output comprises not only hydrocarbons, but also higher alcohols and oxygenates.[^8^, ^9^, ^10^, ^11^] In the past decade, many research efforts were made to revive the more than 100 years old Sabatier reaction, which is the catalytic hydrogenation of CO_2_ towards methane.^[12]^ Ni‐based catalysts are typically used in this process because of their high activity and selectivity, while being inexpensive compared to noble metal‐based catalysts, such as Rh, Ru, Au, and Pt.[^12^, ^13^, ^14^, ^15^, ^16^, ^17^, ^18^, ^19^, ^20^] However, converting CO_2_ towards value‐added products other than methane, using relatively cheap, abundant, and non‐toxic transition metal catalysts (i. e., Fe, Co, Cu) would be a major advance.

Moreover, using waste residues as solid catalysts for CO_2_ conversion towards value‐added products would be of particular interest for the industrial sector. Hereby, on the one hand high costs for waste handling could be minimized, while on the other hand, industrial CO_2_ emissions could be directly converted into value‐added products in a circular fashion. Ideally, gaseous industrial waste streams could directly be used as reactant over the solid process waste products, which then act as solid catalysts for producing value‐added products. To this end, the “recycled process residue catalyst” should be able to convert the waste stream, generally consisting of a mixture of various gases. For example, waste streams from biomass char gasification consist of CO, CO_2_, H_2_, and inert gases.[^21^, ^22^, ^23^, ^24^, ^25^] The solid residue that remains after this process may contain many different elements in various phases and oxidation states. Typically the main components are Si, Al, Ca, Mg, Fe, K, and Na,[^22^, ^24^] but, for example, Ti, S, and P have also been reported.^[23]^ It has already been documented that iron‐containing char from biomass gasification catalyzes hydrocarbon cracking reactions.^[26]^ The ideal solid residue would possess both CO_2_ hydrogenation and Fischer–Tropsch synthesis activity, as the gaseous stream from gasification consists mainly of CO, CO_2_, and H_2_.

For Fischer–Tropsch synthesis (FTS), an industrial process to convert CO and H_2_ into synthetic fuels, Co and Fe are the most widely used catalysts.[^27^, ^28^, ^29^] Cobalt operates at 200–250 °C and mainly yields linear C_5+_ paraffins. Iron, on the other hand, is able to operate in a broader temperature range (200–350 °C) and typically produces more olefins and oxygenates, particularly at higher temperatures (320–350 °C).[^25^, ^30^, ^31^, ^32^] Fischer–Tropsch‐to‐Olefins (FTO), a subclass of the FTS process, is particularly interesting for the direct conversion of alternative carbon resources to lower olefins.^[33]^ For this sustainable process, iron‐based catalysts are preferred over cobalt‐based catalysts because of their high olefin selectivity, low cost, and high water gas shift (WGS) activity. The latter enables the catalyst to alter the H_2_/CO ratio of the syngas,[^32^, ^33^] which is essential when, for example, biomass is used as feedstock and the H_2_/CO ratio of the resulting syngas is below 1.[^25^, ^34^, ^35^] Biomass‐derived syngas may additionally contain CO_2_ and N_2_ since air is typically used as oxidation agent in the biomass gasification process.^[36]^ The specifications of biomass‐derived syngas may lead to low conversion efficiency and worse catalyst performance.^[35]^ Apart from the traditional FTS process, CO_2_ hydrogenation to fuels, also referred to as modified (M)FTS, gained attraction in terms of CO_2_ mitigation strategies.[^37^, ^38^, ^39^, ^40^] Fe‐based FTO catalysts yielded a very stable product selectivity when changing the gas feed from traditional CO and H_2_ mixtures towards CO_2_, CO, and H_2_.[^11^, ^41^, ^42^] Besides, the addition of alkali metals, such as K and Na, to Fe‐based catalysts has been reported to improve long‐chain hydrocarbon and olefin production from CO_2_.[^11^, ^43^, ^44^] Accordingly, solid residue containing Fe and alkali metals might represent a promising candidate for industrial waste stream conversion.

In this work, we have investigated the applicability of a coarse solid residue (CSR) in CO_2_/CO/H_2_ conversion. The CSR catalyst material was generated during a solid waste gasification process at Enerkem (Westbury, Canada). Hereby, we aimed to employ the CSR sample without further modification, potentially enabling the direct usage of industrial solid waste as a suitable solid catalyst to convert gaseous industrial waste streams into valuable products. To evaluate the potential of this CSR sample for CO/CO_2_ hydrogenation, an Fe/SiO_2_ reference catalyst with comparable iron oxide nanoparticle sizes was used, hereby mimicking the main active ingredients of the CSR sample. We show that CSR catalyst materials make methane and olefins from a CO, CO_2_, H_2_, and N_2_ gas feed, thereby mimicking the composition of biomass‐derived syngas. Alkali promoter effects on the CO_2_ and CO hydrogenation performances were investigated using a K−Fe/SiO_2_ reference catalyst. The presence of K enhanced the (reverse) (R)WGS activity and led to an increase in olefin production. Besides catalytic testing, operando X‐ray diffraction (XRD) and Raman spectroscopy studies were performed to gain insights into the catalytically active phase and deducing a fundamental understanding of structure–performance correlations in the CSR samples. It was found that the increased presence of the iron carbide phase in the catalyst materials coincided with an increase in olefin selectivity.

## Results and Discussion

### Chemical composition

The mineral composition of CSR obtained during solid waste gasification, as determined with X‐ray fluorescence (XRF) and inductively coupled plasma ‐ optical emission spectroscopy (ICP‐OES), can be found in Table 1. The main components were the metal oxides SiO_2_, CaO, and Al_2_O_3_, which in heterogeneous catalysis generally function as support or binder material and stabilize the catalytically active metal nanoparticles. Another important component in the CSR sample was Fe_2_O_3_. Fe is believed to be the catalytically active component in solid residues from gasification processes^[26]^ and also the active ingredient in FTO‐based catalyst materials.[^45^, ^46^] Then, the alkali metals Na and K in CSR can act as a promoter or a poison, depending on their concentration and interplay with the active metal phase.[^46^, ^47^, ^48^] As promoters, the alkali metals both increase the reducibility of iron oxides and the carbon deposition rate. The latter is beneficial for FTO, as the active phase is considered an Fe carbide phase.^[45]^ In the CO_2_ hydrogenation reaction, the addition of alkali metals to Fe‐based catalysts has been reported to increase the selectivity towards high‐valued olefins due to RWGS activity.[^43^, ^48^] Moreover, Mg and Ca that are present in CSR have been reported to increase the deactivation rate and the methane formation rate compared to unpromoted and K‐promoted iron‐based FTO catalysts.^[50]^ Besides, CSR contained Cr and Cu, which are known to promote the (R)WGS reaction as well.^[51]^

**Table 1:** CSR chemical composition and loss on ignition (LOI) from various solid waste feedstocks obtained by XRF spectrometry and ICP‐OES^[a]^ analysis of the specific CSR batch used in this study, as well as the Fe/SiO_2_ reference catalyst.

CSR compound	Min. XRF [wt %]	Max. XRF [wt %]	Element	CSR ICP‐OES [wt %]	Fe/SiO_2_ ICP‐OES [wt %]
SiO_2_	38.9	59.2	n. a.	n. a.^[a]^	n. a.^[a]^
CaO	13.0	24.3	Ca	9.5	–
Al_2_O_3_	7.7	36.1	Al	5.1	–
MgO	1.4	3.1	Mg	0.7	–
Na_2_O	1.6	4.2	Na	2.5	–
K_2_O	0.5	1.6	K	1.9	–
MnO	0.1	1.6	Mn	0.04	–
ZrO_2_	0.1	3.0	n.a.	n.a.	–
TiO_2_	0.8	1.5	Ti	0.7	–
Cr_2_O_3_	0	0.1	Cr	0.03	–
Fe_2_O_3_	1.2	4.8	Fe	1.9	7.7
BaO	0	0.1	Ba	0.05	–
SO_3_	0	0.4	S	0.01	–
P_2_O_5_	0	3.0	P	0.7	–
LOI	0	0.2	n.a.	n.a.	–
			Cu	0.2	–

[a] The Si concentration could not be determined quantitatively with ICP‐OES. LOI: Loss on ignition.

### Morphology of the coarse solid residue sample

**Figure 1:**
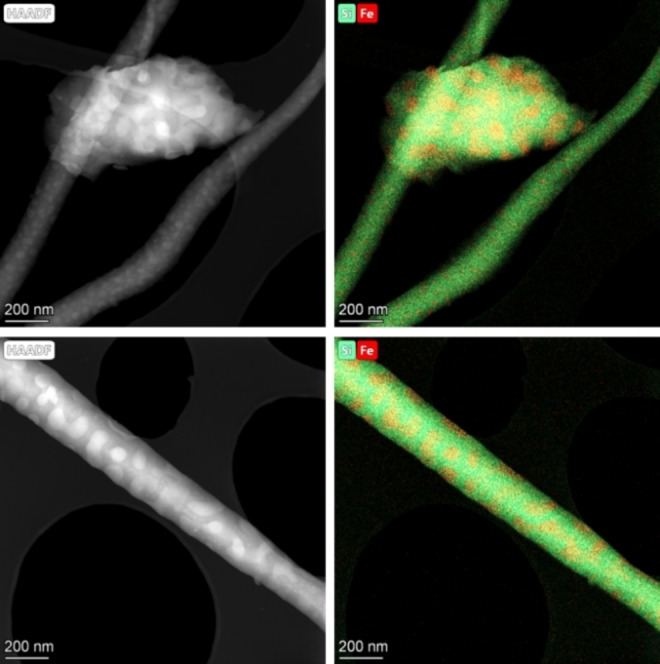
HAADF‐STEM images of the fresh CSR sample (left) and EDX chemical mapping (right). Fe is shown in red, and Si is shown in green.

The morphology of the CSR sample and spatial distribution of the elements were investigated with electron microscopy (EM) and energy‐dispersive X‐ray spectroscopy (EDX). The CSR morphology resembled a typical Fe/SiO_2_ heterogeneous catalyst: Fe nanoparticles supported by a SiO_2_ matrix (Figure 1). However, CSR contained more elements than just Fe and Si. For example, Al appeared to be in the same location as Si, whereas Ca and Ti appeared to be in close vicinity of the Fe nanoparticles (Figures S1 and S2). The average Fe_2_O_3_ nanoparticle size was 64±16 nm from the high‐angle annular dark‐field (HAADF) scanning transmission electron microscopy (STEM) images (Figure S3).

### Crystalline phases and Fe crystallite sizes

**Figure 2:**
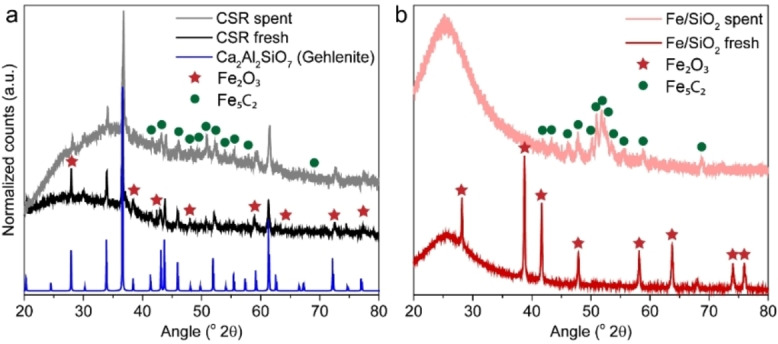
Characterization of the crystalline phases in the CSR sample and a reference Fe/SiO_2_ catalyst material with XRD. (a) XRD patterns of the fresh and spent (*T*=450 °C, *P*=5 bar, and CO/CO_2_/H_2_/N_2_=4.5 : 2.5 : 3 : 1) CSR sample. XRD pattern of the mineral gehlenite from the PDF‐4+ XRD database is added as a reference. (b) Fe/SiO_2_ (7.7 wt %) fresh and spent (*T*=450 °C, *P*=5 bar, and CO/CO_2_/H_2_/N_2_=4.5 : 2.5 : 3 : 1).

The crystalline phases and Fe crystallite sizes in the CSR sample and Fe/SiO_2_ reference catalyst were analyzed with XRD (Figures 2 and S4). The diffraction peaks of the CSR sample matched with the mineral gehlenite (Ca_2_Al_2_SiO_7_) and with hematite (Fe_2_O_3_). For the Fe/SiO_2_ reference catalyst only hematite (Fe_2_O_3_) was detected as crystalline phase. The average Fe_2_O_3_ crystallite size was 63 nm for the CSR sample and 53 nm for the Fe/SiO_2_ reference catalyst.

### C_2+_ hydrocarbon production from CO/CO_2_/H_2_ mixtures

**Figure 3:**
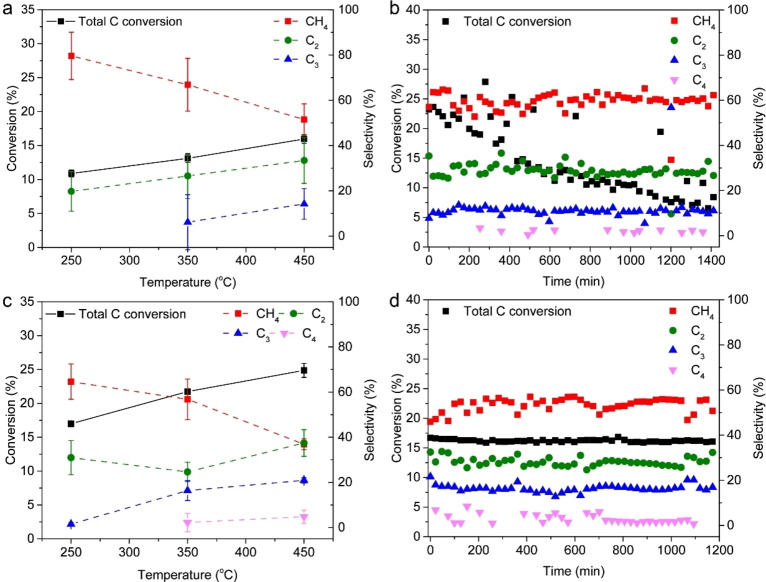
Catalytic performance of the CSR sample and the Fe/SiO_2_ reference catalyst material. Catalytic testing of (a) CSR and (c) Fe/SiO_2_ in CO/CO_2_/H_2_/N_2_=4.5 : 2.5 : 3 : 1 at *P*=5 bar, *T*=250, 350, 450 °C, and gas hourly space velocity (GHSV)=3400 h^−1^ (12 h per temperature). Stability testing of (b) CSR and (d) Fe/SiO_2_ at 450 °C for 24 and 20 h, respectively, under the same gases and pressure as (a).

The CSR sample and the Fe/SiO_2_ reference catalyst were catalytically tested in CO/CO_2_/H_2_/N_2_=4.5 : 2.5 : 3 : 1 and at 5 bar pressure (Figure 3). This particular gas feed composition was chosen with renewable energy resources in mind, as biomass‐derived syngas typically has a gas composition with H_2_/CO<1 and may contain CO_2_ and N_2_.[^25^, ^34^] Prior to the reaction, the sample was heated in H_2_ at 450 °C to transform Fe_2_O_3_ into metallic Fe. The reduction profiles of CSR and Fe/SiO_2_ can be found in Figure S5. First, we explored the influence of reaction temperature by performing multiple tests at 250, 350, and 450 °C (Figure 3a, c). We found 450 °C as the optimum operating temperature for the CSR sample (Figure 3a), as the total carbon conversion increased with temperature. Besides, the very exothermic methane formation reactions from CO (Δ*H*=−220 kJ mol^−1^ at 450 °C) and CO_2_ (Δ*H*=−183 kJ mol^−1^ at 450 °C) are thermodynamically more favorable at relatively low temperatures (see also thermodynamic calculations in Figure S6) and in high H_2_ concentrations. This was indeed reflected by the methane selectivity, which was the lowest at 450 °C. From CO and H_2_ the C_2_ and C_3_ olefin formation reactions are exothermic, while from CO_2_ and H_2_ these reactions are endothermic.^[49]^ In our study, higher temperature appeared favorable for the lower olefin yield (Tables S1 and S2) from the CO/CO_2_/H_2_/N_2_=4.5 : 2.5 : 3 : 1 gas feed; propene was only formed at 350 and 450 °C and not at lower temperatures. Compared to the total carbon conversion of the Fe/SiO_2_ reference catalyst (24.9 % at 450 °C), CSR had a lower overall carbon conversion (16.0 % at 450 °C) (Tables S1–S4). The lower carbon conversion of CSR compared to the Fe/SiO_2_ reference catalyst is explained by the lower content of the active ingredient Fe in CSR (1.9 wt %) compared to Fe/SiO_2_ (7.7 wt %), as displayed in Table 1. Besides, the high content of K (1.9 wt %) and Na (2.5 wt %) in CSR could have a detrimental effect on catalytic performance.^[47]^ Moreover, Mg and Ca in CSR could increase the deactivation rate and the methane formation rate during the FTO reaction.^[50]^

**Figure 4:**
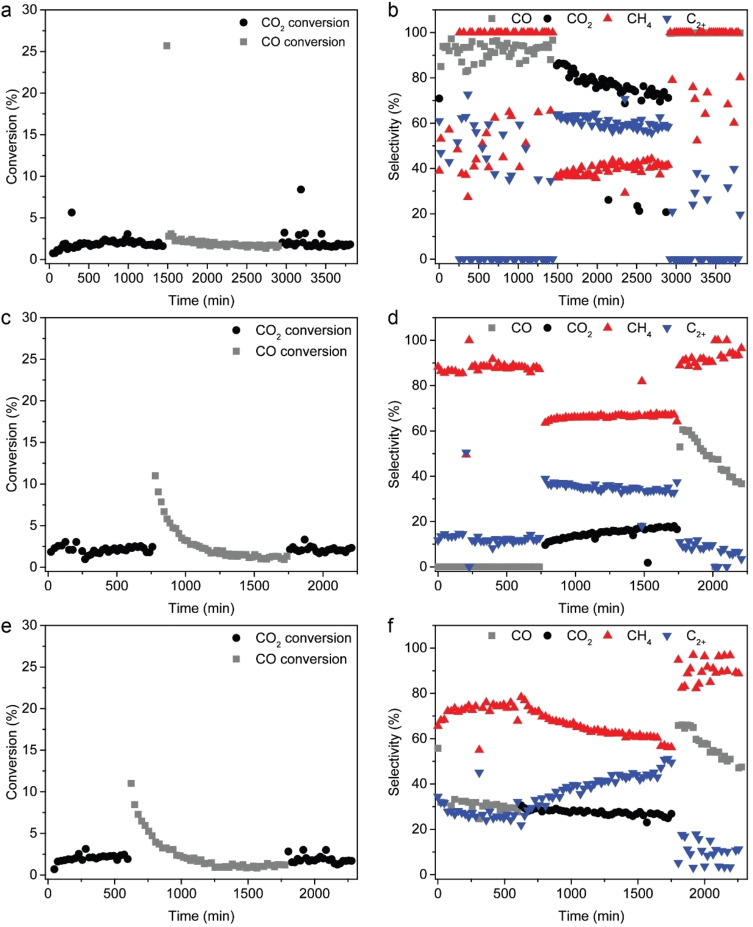
Catalytic performance in (consecutively) CO_2_ hydrogenation reaction, FTO reaction, and subsequent CO_2_ hydrogenation reaction of (a,b) CSR, (c,d) Fe/SiO_2_, and (e,f) K−Fe/SiO_2_. The CO_2_ hydrogenation steps were carried out at *T*=250 °C, *P*=5 bar, H_2_/CO_2_=3, GHSV=3070 h^−1^, while the FTO step was carried out at *T*=350 °C, *P*=5 bar, H_2_/CO=0.7, GHSV=2425 h^−1^. The hydrocarbon (CH_4_ and C_2+_) selectivities displayed are CO and/or CO_2_‐free. Prior to the first CO_2_ hydrogenation step, the samples were pre‐reduced at 450 °C in N_2_/H_2_=2 for 1 h.

The stabilities of the CSR sample and Fe/SiO_2_ were evaluated in CO/CO_2_/H_2_/N_2_=4.5 : 2.5 : 3 : 1, *P*=5 bar, and *T*=450 °C (see Figure 3b, d and Tables S2 and S4). For CSR, the methane (59.3 %), C_2_ (28.6 %), and C_3_ (11.6 %) selectivities remained stable over the course of 24 h. In some of the gas chromatography (GC) injections, C_4_ products were detected as well (Figure 4b and Table S2; 0.5 % average selectivity). However, the total carbon conversion decreased over time. Considering the high CO/CO_2_ ratio (CO/CO_2_=1.8) in the gas feed, the occurrence of the WGS reaction (CO+H_2_O→CO_2_+H_2_; Δ*H*=−37.8 kJ mol^−1^ at 450 °C) could provide a potential explanation for the decrease in carbon conversion. Fe‐based catalysts are known to promote this reaction at moderately high temperatures (350–500 °C).^[51]^ Besides, CSR contains Cr, Cu, K, and Na, which are known to promote the WGS reaction as well.^[51]^ CO and the inevitably formed H_2_O then produce CO_2_, which is less reactive than CO and consequently lowers the total carbon conversion. Besides, Mg and Ca, also present in CSR, have been reported to increase the deactivation rate during the FTO reaction.^[50]^ An alternative explanation for the lower carbon conversion could be the oxidation of iron phases on the catalyst surface. CO_2_ and H_2_O are known to have an oxidizing effect and thereby deactivate conventional iron‐based catalysts.[^25^, ^32^, ^52^]

### Fe carbides in the spent catalyst materials

The spent CSR sample as well as the Fe/SiO_2_ reference catalyst material contained Fe carbides as determined with XRD (Figure 2). The Hägg carbide (Fe_5_C_2_) is known to be the most stable Fe carbide phase under FTO reaction conditions,^[45]^ and this was the only Fe carbide phase observed in this study. Fe_5_C_2_ has also been proposed as main active phase in the FTO reaction and postulated as responsible phase for hydrocarbon chain growth.^[53]^ To further investigate the correlations between alkali promoter elements, (R)WGS activity, Fe carbide formation, and catalytic performance, CSR, Fe/SiO_2_, and K−Fe/SiO_2_ were tested consecutively for CO_2_ hydrogenation, FTO, and again CO_2_ hydrogenation to assess whether iron carbide formation affected the RWGS activity.

### Reverse water gas shift activity promoted by alkali elements

To examine the occurrence of the RWGS reaction, we tested the CSR sample, the Fe/SiO_2_ reference catalyst material, and a K‐promoted Fe/SiO_2_ reference catalyst material [0.71 wt % K and 7.6 wt % Fe (ICP‐OES)] for CO_2_ hydrogenation. Alkali elements, like K, are known to promote the (R)WGS reaction,^[51]^ leading to an increased CO selectivity during CO_2_ hydrogenation, which could boost the overall catalyst performance. First, the samples were pre‐reduced at 450 °C in N_2_/H_2_=2 for 1 h. Then, the CO_2_ hydrogenation performance was tested for at *T*=250 °C, *P*=5 bar, and H_2_/CO_2_=3. Subsequently, the FTO performance was tested at *T*=350 °C, *P*=5 bar, and H_2_/CO=0.7. Thereafter, the samples were again tested for CO_2_ hydrogenation performance to investigate whether iron carbide formation, known to occur in the presence of H_2_‐poor syngas,[^45^, ^46^] affected RWGS activity. As can be seen in Figure 4, relatively low CO_2_ conversions were observed at 250 °C for all samples (≈2 %). Interestingly, CSR displayed high RWGS activity and produced 91.9 % CO during the first CO_2_ hydrogenation step (Figure 4a, b). The Fe/SiO_2_ catalyst did not produce any CO and appeared thus inactive for the RWGS reaction (Figure 4c, d). However, the presence of the alkali element K promoted the RWGS activity drastically, as the K−Fe/SiO_2_ catalyst produced 30.4 % CO (Figure 4e, f). The RWGS activity induced by K was beneficial for C_2+_ production, as the C_2+_ selectivity increased from 12.9 % with Fe/SiO_2_ to 28.2 % with K−Fe/SiO_2_ (CO‐free selectivities). Besides, the C_2_–C_4_ hydrocarbons produced by K−Fe/SiO_2_ contained more olefins compared to unpromoted Fe/SiO_2_. The complete product distribution as well as olefin/paraffin ratios can be found in Tables S5 and S6. Increased C_2+_ production and olefin selectivity by iron‐based catalysts upon K promotion has been reported previously for CO_2_ hydrogenation.[^25^, ^43^]

During the FTO step, the initial apparent CO conversions were high (≈11–25 %) for all samples (Figure 4), likely due to CO consumption for iron carbide formation. Under FTO conditions, carbon diffusion into iron has a lower activation barrier than the FTO reaction.^[31]^ Hence, CO will mostly be used for iron carbide formation until a saturated metal carbide is formed. For Fe/SiO_2_, the catalyst performance deteriorated slightly over time, as more methane and CO_2_ were produced (Figure 4c, d). K−Fe/SiO_2_ (Figure 4e, f), on the other hand, produced less methane and CO_2_, while more C_2+_ was formed over time. For CSR (Figure 4a, b), the CO_2_ selectivity went down, the methane selectivity became slightly higher, and the C_2+_ selectivity slightly lower over time (Tables S5 and S6). Alkali elements thus appeared to limit catalyst deactivation during the FTO reaction. Besides, alkali promoters clearly enhanced the WGS activity, as average CO_2_ selectivities of 14.6, 27.5, and 73.1 % were observed for Fe/SiO_2_, K−Fe/SiO_2_, and CSR, respectively. The C_2+_ selectivity again seemed to benefit from K promotion: On average Fe/SiO_2_ produced 34.5 % C_2+_ (Figure 4d), while K−Fe/SiO_2_ displayed 40.1 % C_2+_ hydrocarbons (Figure 4f), and CSR 60.5 % (Figure 4b) CO_2_‐free selectivities). The C_2+_ hydrocarbons produced by K−Fe/SiO_2_ and CSR contained more olefins compared to unpromoted Fe/SiO_2_.

In the second, consecutive, CO_2_ hydrogenation step, higher methane and CO selectivities were observed compared to the first CO_2_ hydrogenation step for all samples. For CSR, 99.4 % CO was observed (Figure 4b), while Fe/SiO_2_ displayed 48.0 % (Figure 4d) and K−Fe/SiO_2_ 58.1 % (Figure 4f). The iron carbide phase, as formed under FTO conditions (Figures 2 and S7), thus appeared to have a higher RWGS activity compared to the metallic iron phase present after reduction. These consecutive CO_2_ hydrogenation/FTO/CO_2_ hydrogenation experiments were additionally conducted at 450 °C for CSR and Fe/SiO_2_ (Table S7). Although the CO_2_ and CO conversions were higher at 450 °C than at 250 or 350 °C, the general conclusions as drawn above also applied at higher temperature.

### Fe carbides evolved as active phase in the coarse solid residue sample under reaction conditions

**Figure 5:**
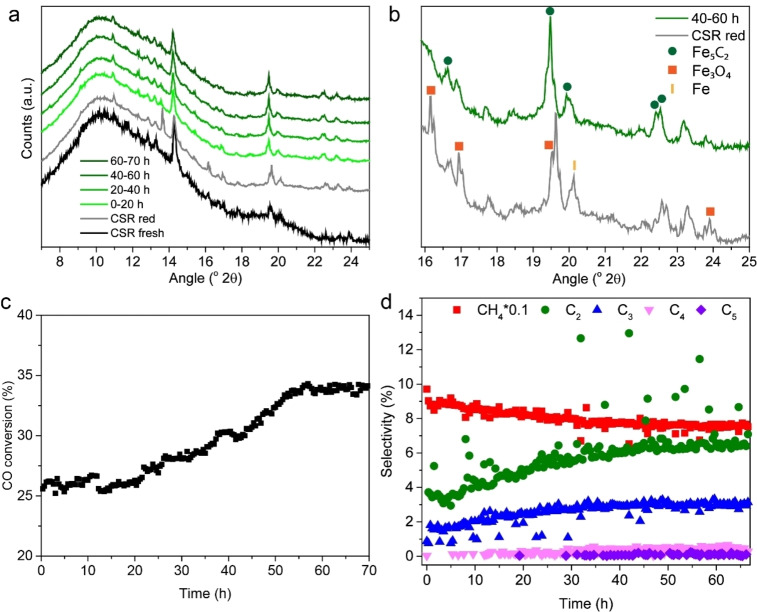
Operando XRD of the CSR sample. (a) CSR fresh, after reduction at 450 °C in H_2_, and during CO hydrogenation (H_2_/CO=0.7) for 70 h at 450 °C and 5 bar. (b) Zoom in of 16–25 ° 2θ, showing CSR contained a mixture of Fe_3_O_4_ and metallic Fe after reduction. Under reaction conditions (Fe_5_C_2_), Hägg carbide, evolved as active phase in the CSR sample. (c) CO conversion [%] and (d) product selectivities over time.

To further investigate the formation of iron carbides, operando XRD was performed under FTO conditions. Operando XRD patterns of the CSR sample were recorded after reduction at 450 °C in H_2_, and during CO hydrogenation (H_2_/CO=0.7) for 70 h at 450 °C and 5 bar (see Figure 5). After the reduction procedure, the CSR sample contained a mixture of Fe_3_O_4_ and metallic Fe (Figure 5a, b). Under H_2_‐poor FTO reaction conditions this slowly transformed into the Hägg carbide phase (Fe_5_C_2_) (Figure 5b), which correlated with an increase in CO conversion and an increase in C_2+_ selectivity (Figure 5c, d). C_5_ products were only detected after around 30 h time‐on‐stream. Both the CO conversion and the C_2+_ selectivity reached a stable level after about 55 h time‐on‐stream. The olefin/paraffin ratio in the C_2_–C_4_ products increased with increasing reaction time, suggesting that the Fe_5_C_2_ phase is more selective to olefins compared to metallic Fe. A complete overview of all the products detected, including all isomers, can be found in Table S8. At 40–60 h time‐on‐stream (green XRD pattern in Figure 5b), CSR solely contained the Hägg carbide phase (Fe_5_C_2_), while Fe_3_O_4_ and metallic Fe were not observed. We can thus conclude that Hägg carbide is the (most) active phase for C_2+_ production, as its emergence was correlated with enhanced catalytic performance. This is in accordance with an earlier study that ascribed the increase in C_2+_ selectivity to the transition of metallic iron to iron carbides.^[54]^

### Improving C_2+_ selectivity with increased reaction pressure

**Figure 6:**
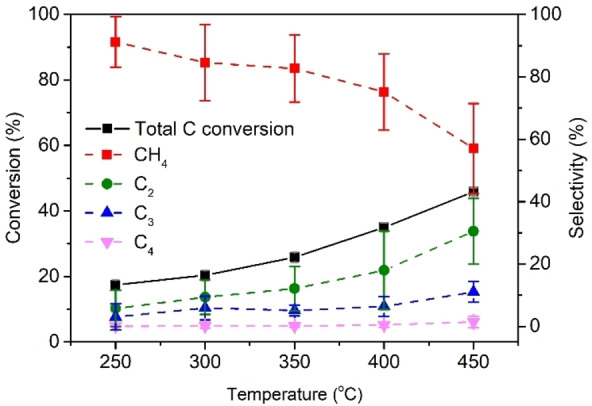
Catalytic performance of the CSR sample in CO/CO_2_/H_2_/N_2_=4.5 : 2.5 : 3 : 1 at *P*=20 bar, *T*=250–450 °C, GHSV=3400 h^−1^ (6 h per temperature).

To improve the selectivity to lower olefins, we increased the pressure to 20 bar and tested the CSR sample at different temperatures (250–450 °C) (Figure 6). Again, 450 °C appeared to be the optimum temperature for CO/CO_2_ conversion (total C conversion=45.9 %) and C_2+_ production. At 20 bar the methane selectivity went down to 57.1 % and the C_2_–C_4_ olefin selectivity increased up to 42.9 %. The olefin/paraffin ratio increased with pressure as well (Table S9). Even though the CSR sample may not outperform commercial Fe‐based FTO catalysts (CH_4_ selectivity=3–42 %^[30]^) or Fe‐based catalysts developed for CO_2_ to fuels (CH_4_ selectivity=3–16 %[^11^, ^43^]), our results present an attractive strategy in the field of renewables.[^25^, ^55^] Fe‐containing CSR catalyzes the conversion of CO/CO_2_ to valuable olefins and would otherwise have been discarded as an industrial waste product. Repurposing the waste product as a CO/CO_2_ conversion catalyst represents an example of a strategy to reuse and thus minimize industrial waste streams.

### Carbonaceous deposits evolved under optimized reaction conditions

**Figure 7:**
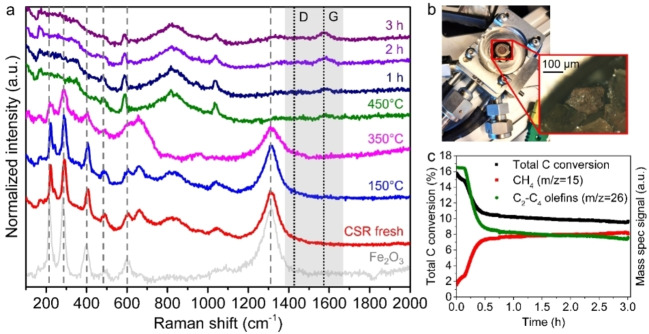
Operando Raman micro‐spectroscopy on the CSR sample (a) during the reduction procedure in H_2_/Ar=1 and during CO/CO_2_ hydrogenation (CO/CO_2_/H_2_=2.2 : 1.2 : 1.5) at *P*=20 bar and *T*=450 °C. The Fe_2_O_3_ peaks are indicated with a gray dashed lines and the carbon D and G bands with black dotted lines. (b) Photograph of the high‐pressure Raman cell and microscopy image of a CSR particle. (c) MS signals for CH_4_ (m/z=15) and C_2_–C_4_ olefins (m/z=26).

To investigate whether or not carbon deposits were formed under reaction conditions, we have performed operando Raman micro‐spectroscopy studies (Figure 7a, b). While X‐ray diffraction techniques, like XRD, are usually the method of choice for crystalline solid materials (i. e., long‐range order), Raman spectroscopy is more promising to analyze carbon deposits with a highly disordered structure.[^56^, ^57^] Raman spectroscopy probes molecular structures (short‐range order) and is sensitive to the degree of structural disorder. We compared the fresh CSR sample with an Fe_2_O_3_ (hematite) reference. CSR indeed displayed the characteristic Fe_2_O_3_ peaks at 222, 242, 291, 408, 490, 608, 662, and 1309 cm^−1^ (Figure 7a).^[58]^ Structural changes were not yet observed at 150 °C in H_2_. However, at 350 °C line broadening was visible, indicating that Fe_2_O_3_ was transformed into Fe_3_O_4_ (magnetite). At 450 °C, the iron oxide peaks had disappeared, indicating the presence of the Raman‐inactive metallic iron. Then, CO/CO_2_ hydrogenation was carried out at 20 bar and 450 °C. After 1–3 h of reaction, bands at 1340 and 1580 cm^−1^ appeared, indicating the formation of carbonaceous species.[^54^, ^58^] This is in line with earlier studies on Fe‐based FTO catalysts, where carbonaceous species were observed during CO hydrogenation.^[45]^ Inactive carbon species could play a role in catalyst deactivation,^[32]^ but carbonaceous species have also been reported as intermediates in CO hydrogenation processes.[^54^, ^58^] While carbonaceous species evolved during our experiments, the total carbon conversion, as measured (semi‐quantitatively) with mass spectrometry (MS) and compared to a blank measurement, decreased. Besides, the MS response of CH_4_ increased, while the C_2_–C_4_ olefins MS response (Figure 7c) decreased over time. Although the deposition of carbonaceous species coincided with deteriorating catalytic performance, the apparent catalyst deactivation could also be assigned to other causes. For example, the presence of Mg and Ca has previously been reported to increase catalyst deactivation and methane formation.^[50]^ Moreover, the presence of K, Na, Cr, and Cu in CSR promote the WGS reaction,^[51]^ which could increase the amount of CO_2_ in the gas feed and consequently decrease the catalytic performance. Re‐oxidation of iron (carbide) nanoparticles was, however, not observed in the Raman spectra.

## Conclusions

A coarse solid residue (CSR) material, which is a waste product obtained from solid waste gasification, was tested as a potential solid catalyst for CO and CO_2_ hydrogenation. Fe carbides were identified as active phase during CO/CO_2_ conversion with operando X‐ray diffraction and were linked to an increase in CO/CO_2_ conversion and the desired light olefin selectivity. More specifically, the CSR material produced 57 % methane and 43 % C_2_–C_4_ olefins from a CO/CO_2_/H_2_ mixture with a total C conversion of 46 % at *T*=450 °C and *P*=20 bar. The alkali elements in the CSR material appeared responsible for the (reverse) water gas shift activity and for an increased C_2+_ olefin production. With these new insights, the gasification process conditions could be optimized to obtain a catalytically superior CSR material, for example, with a higher Fe content. Strategies to reuse waste streams, like the one presented in this work, should be widely employed to minimize industrial waste output. Besides, recycling waste streams will decrease the usage of valuable raw materials required for, for example, catalyst synthesis. Considering that the CSR material used catalyzes the conversion of CO/CO_2_ mixtures into valuable olefins and would otherwise have been discarded as industrial waste, our findings offer a new perspective on how waste streams can be utilized. In this manner, it adds to the concept of materials circularity and related metal scarcity abatement.

## Experimental Section

### Catalyst preparation

The industrial waste sample, a coarse solid residue (CSR) sample, also called “slag”, was generated during solid waste residue gasification process at Enerkem (Westbury, Canada). The CSR sample was used without further treatment. The Fe/SiO_2_ (7.7 wt % Fe) and the K−Fe/SiO_2_ (0.71 wt % K and 7.6 wt % Fe) reference catalysts were prepared by the incipient wetness impregnation (IWI) technique. To this purpose, commercially available high‐purity grade silica gel (Davisil Grade 643, pore size 150 Å, 200–425 mesh, Sigma‐Aldrich) was used as support material. The synthesis procedure was conducted as follows. Initially, the required amount of FeCl_3_ ⋅ 6 H_2_O (Sigma‐Aldrich, ≥99 %) was dissolved in water, whereby the volume of water was adjusted to that of the pore volume of the silica support. After impregnation and drying at 60 °C for 24 h, calcination of the impregnated catalyst was conducted under flowing N_2_ (100 mL min^−1^) in a tubular furnace at 450 °C for 5 h (5 °C min^−1^ ramp). The K‐promoted Fe/SiO_2_ catalyst was prepared by consecutive impregnation with an aqueous solution of K_2_CO_3_ (Sigma‐Aldrich, ≥99 %), drying, and calcination as described above.

### Catalyst characterization

**Elemental composition**: The metal concentrations were determined via ICP‐OES with a PerkinElmer Avio^®^ 500 ICP Optical Emission Spectrometer. Here, the CSR and Fe/SiO_2_ samples were prepared by iron extraction in aqua regia. Additionally, the chemical composition of the CSR sample was analyzed by XRF spectrometry (Panalytical, Axios Advanced).

**Transmission electron microscopy**: Electron microscopy investigations were performed in transmission mode and HAADF using a FEI Talos F200X microscope operating at 200 kV. Elemental mapping was performed using EDX. For the TEM measurements, the samples were suspended in ethanol under ultrasonic vibrations. Subsequently, a drop of the suspension was deposited onto a Carbon‐type B copper 200 mesh grid. For determining the particle size distribution of the supported nanoparticles from TEM images, the software ImageJ was used for manually fitting the particle diameters (>100 particles).

**Ex‐situ X‐ray diffraction**: Ex‐situ XRD patterns of the fresh and spent catalysts were recorded on a Bruker D2 Phaser X‐ray diffractometer using Co K_α12_ radiation (*λ*=1.790 Å) in the range of 2*θ*=20–80° with a scan step size of 0.01° and scan time 1 s per step. The Fe_2_O_3_ average crystallite sizes were estimated by applying the Scherrer equation (k‐factor of 0.9) to the (012) diffraction of Fe_2_O_3_ (2*θ*=28.0°).

**Operando X‐ray diffraction**: Operando XRD patterns were recorded on a Bruker D8 Discover X‐ray diffractometer in Debye–Scherrer transmission (capillary) geometry with a Mo (K_α1_=0.709 Å) source was used. At the beginning of each operando reaction run, the capillary was moved to the focus of the X‐ray beam (beam≈600×15 000 μm, height×width) for maximum diffraction. The XRD patterns were collected over a 2*θ* range of 7–25° with a scan step size of 0.015°. Data were collected of the fresh sample, during reduction at 450 °C in pure H_2_ (3 mL min^−1^), after reduction, and during CO hydrogenation (CO/H_2_/He=2.25 : 1.5 : 1). The products were analyzed with on‐line GC (Thermo Fischer Scientific).

**Operando Raman micro‐spectroscopy**: Raman spectra were recorded using a Horiba Xplora with a 532 nm laser and 1200 grating for 30 s with 5 accumulations. For operando experiments, Raman high temperature reaction chamber from Harrick Scientific, suitable for high pressures and temperatures, was used. To monitor the iron phase during the reduction procedure, the CSR sample was heated to 450 °C with 10 °C min^−1^ in 3 mL min^−1^ H_2_ and 3 mL min^−1^ Ar. Then, the sample was exposed to 2.2 mL min^−1^ CO, 1.2 mL min^−1^ CO_2_, 1.5 mL min H_2_, and 0.2 mL min^−1^ Ar at *P*=20 bar and *T*=450 °C. Meanwhile, Raman spectra were recorded to monitor the iron phase and carbonaceous species under reaction conditions. The gaseous products were analyzed with on‐line MS (Pfeiffer Vacuum).

### Catalytic performance

Catalyst performance was tested in a fixed‐bed reactor. The steel reactor was typically filled with 200–250 mg of catalyst sample sieved to a grain size of 150–425 μm. The sample was plugged between two quartz wool plugs. The reactor was placed in an oven and was connected to the gas inlet and outlet. A back pressure controller (BPC) was incorporated in the gas line connected to the outlet to maintain a defined pressure. An on‐line gas Thermo Fischer Trace 1300 GC was used for product analysis. The catalyst was reduced in 20 mL min^−1^ H_2_ and 40 mL min^−1^ N_2_ at 450 °C for 1 h (10 °C min^−1^ ramp). Then, the reactor was cooled down to 250 °C with a 10 °C min^−1^ ramp in the same atmosphere. At 250 °C the gas flow was switched to 22.5 mL min^−1^ CO, 12.5 mL min^−1^ CO_2_, 15 mL min^−1^ H_2_, 5 mL min^−1^ N_2_, and 1.2 mL min^−1^ Ar. The pressure was built up with 1 bar min^−1^ to 5 bar or 20 bar. For 12–24 h the products of the reaction were analyzed with an on‐line GC (injection every 23 min). This was repeated at 300–450 °C. The conversion and selectivities were calculated from the obtained GC data. The amounts of converted CO or CO_2_ were calculated using Equation 1:

(1) $$X_{CO}\;\left[\%\right]=\left(1-\frac{A_{CO}/A_{Ar}}{A_{CO}^{0}/A_{Ar}^{0}}\right)\times 100\;\%$$

*A*_CO_ and *A*_Ar_ represent the thermal conductivity detector (TCD) peak area of CO and Ar during the reaction. *A* ^*0*^_CO_ and *A* ^*0*^_Ar_ are the TCD peak areas of CO and Ar recorded during a blank measurement. The selectivity was calculated using Equation 2:

(2) $$S_{i}\;\left[\%\right]=\left(\frac{A_{i}\times \;F_{i}}{\sum A_{i}\times \;F_{i}}\right)\times 100\;\%$$

In this equation, *A*_i_ corresponds to the peak area of product *i*, and *F*_i_ represents the response factor of the analyte.^[59]^

## Conflict of interest

The authors declare no conflict of interest.

1

## Supporting information

As a service to our authors and readers, this journal provides supporting information supplied by the authors. Such materials are peer reviewed and may be re‐organized for online delivery, but are not copy‐edited or typeset. Technical support issues arising from supporting information (other than missing files) should be addressed to the authors.

Supporting Information

## Data Availability

The data that support the findings of this study are available from the corresponding author upon reasonable request.
