# Supplementary material for: Using Biomass Gasification Mineral Residue as Catalyst to Produce Light Olefins from CO, CO2, and H2 Mixtures
Source: ChemSusChem. 2022 Mar 28;15(11):e202200436. doi: 10.1002/cssc.202200436 (PMC9314133; doi:10.1002/cssc.202200436)
Supplement: Supplementary file 1 — Supporting Information [file CSSC-15-0-s001.pdf]

ChemSusChem

Supporting Information

Using Biomass Gasification Mineral Residue as Catalyst to Produce Light Olefins from CO, CO_2, and H_2 Mixtures

Iris C. ten Have, Robin Y. van den Brink, Stéphane C. Marie-Rose, Florian Meirer,* and Bert M. Weckhuysen*© 2022 The Authors. ChemSusChem published by Wiley-VCH GmbH. This is an open access article under the terms of the Creative Commons Attribution License, which permits use, distribution and reproduction in any medium, provided the original work is properly cited.

1. Experimental Section

Scanning electron microscopy. Electron microscopy investigations were performed in scanning mode (SEM) with energy dispersive X-ray (EDX) spectroscopy using a FEI Helios Nanolab.G3 operating at 5-30 keV.

H_2-temperature-programmed reduction. Temperature-programmed reduction (TPR) measurements were performed using a Micromeritics AutoChem II 2920. Samples were placed on quartz wool into a U-tube quartz reactor. The gas mixture consisted of 5% H_2 in Ar with a total gas flow of 40 ml/min. H_2 TPR was carried out by heating with 5°C/min up to 800°C for Fe/SiO_2 catalysts and the coarse solid residue (CSR) sample and held for 30 min at this temperature. A constant initial sample weight of 0.05 g was used and H_2 consumption was continuously monitored by a thermal conductivity detector.

2. Additional Results and Related Discussions

2.1. Catalyst Materials Characterization

SEM-EDX showed large heterogeneity in the CSR sample (**Figure S1**). High-angle annular dark-field (HAADF)-scanning transmission electron microscopy (STEM) and EDX mapping (**Figure S2**) showed the spatial distribution of the elements Si, Fe, K, Na, Ca, Al, Mg, Ti, and O. Si and Al appeared to function as support materials for the Fe nanoparticles. On the other hand, Ca and Ti appeared in the same location as Fe. K, Na, Mg, and O seemed homogeneously distributed. The average Fe_2O_3 nanoparticle size was 64 ± 16 nm from the HAADF-STEM images (**Figure S3**).

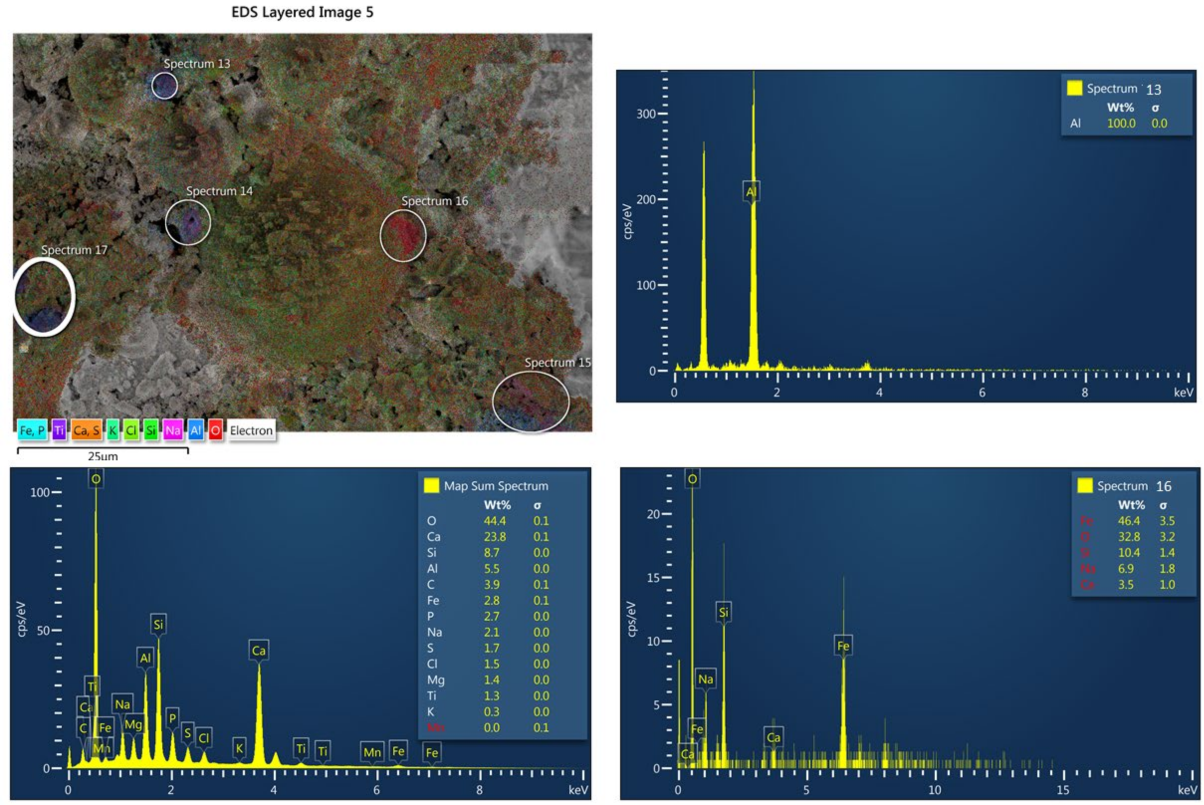

Figure S1. Scanning electron microscopy (SEM) with energy-dispersive X-ray spectroscopy (EDX) image (top left) and the corresponding map sum spectrum (bottom left). Spectrum 13 (top right) was exclusively aluminum oxide, while spectrum 16 contained mostly iron oxide (bottom right).

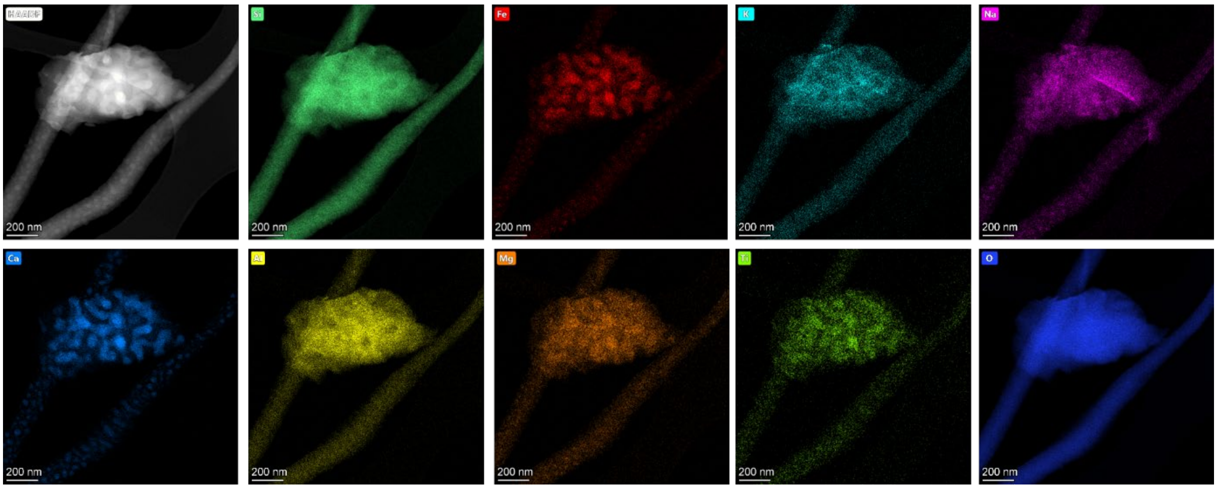

Figure S2. High-angle annular dark-field (HAADF)-scanning transmission electron microscopy (STEM) image (top left) and energy dispersive X-ray spectroscopy (EDX) chemical mapping of Si, Fe, K, Na, Ca, Al, Mg, Ti, and O.

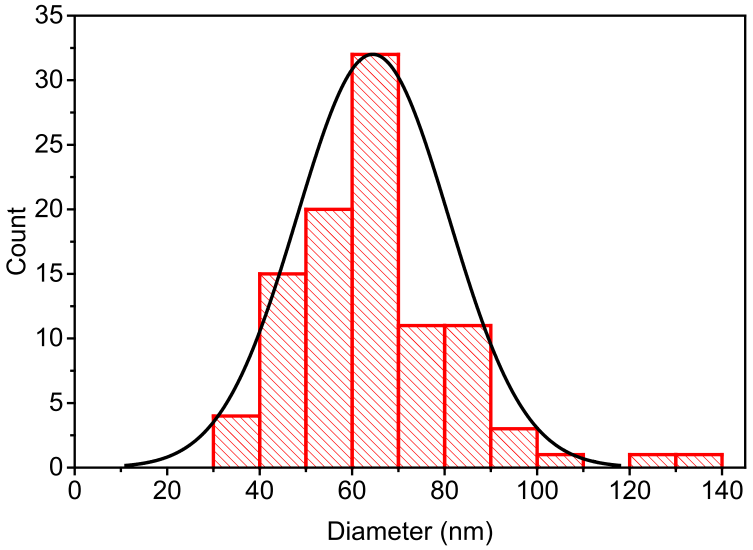

Figure S3. Particle size distribution of the Fe_2O_3 nanoparticles in the CSR sample. The average Fe_2O_3 nanoparticle size was 64 ± 16 nm and in total 100 particles were counted from the STEM-HAADF images.

Figure S4 shows X-ray diffraction patterns of references from the PDF-4+ X-ray diffraction (XRD) database used in this study.

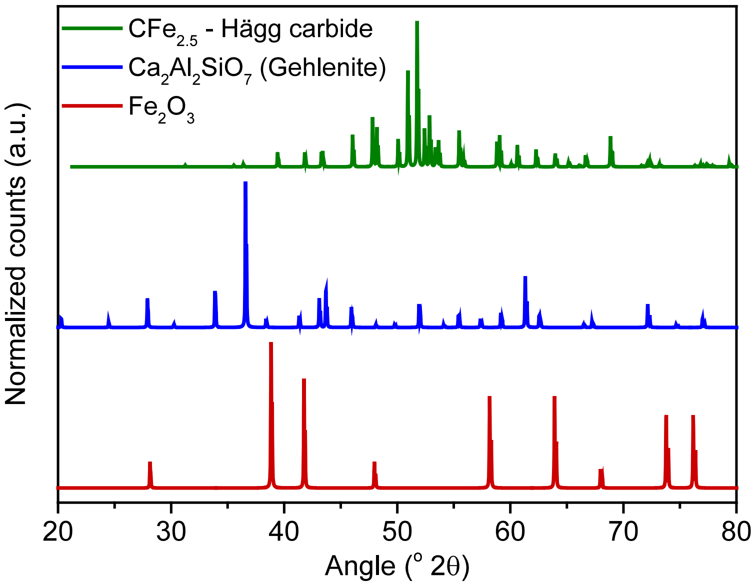

Figure S4. X-ray diffraction (XRD) patterns from the PDF-4+ XRD database.

H_2-TPR was used to follow the reduction of Fe_2O_3. Typically, Fe_2O_3 (hematite) reduces first to Fe_3O_4 (magnetite) and then to metallic Fe. This was indeed apparent in the TPR data of the Fe/SiO_2 reference catalyst (red line in **Figure S5**), where 2 peaks were observed around 500 and 600°C for Fe_2O_3 to Fe_3O_4 and Fe_3O_4 to Fe^0. For the CSR sample, the Fe_2O_3 to Fe_3O_4 transition takes place already at 350°C and the Fe_3O_4 to Fe^0 transition appears as a shoulder around 450°C.

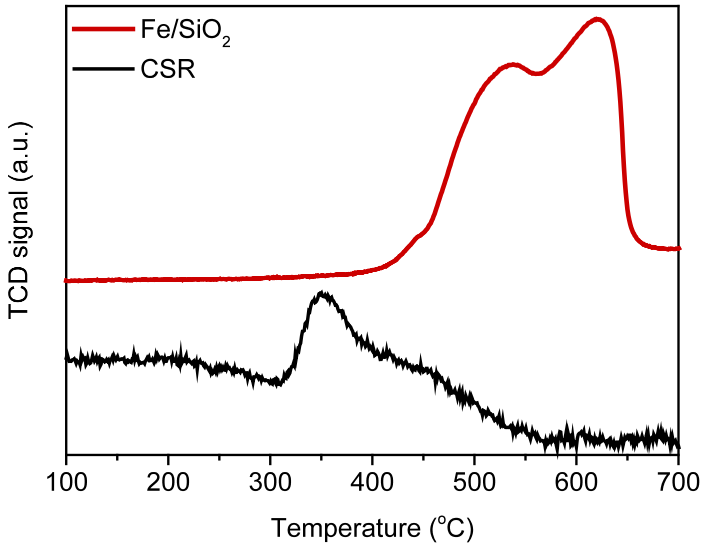

Figure S5. H_2-temperature-programmed reduction (TPR) profiles for the Fe/SiO_2 reference catalyst and for the CSR sample.

2.2. Thermodynamic Calculations

Thermodynamic calculations were performed using HSC chemistry 9. The Gibbs free energies and the reaction enthalpies were calculated from 0 to 1000°C with 50°C step size for the reactions possible during the CO/CO_2 hydrogenation with the CSR sample.

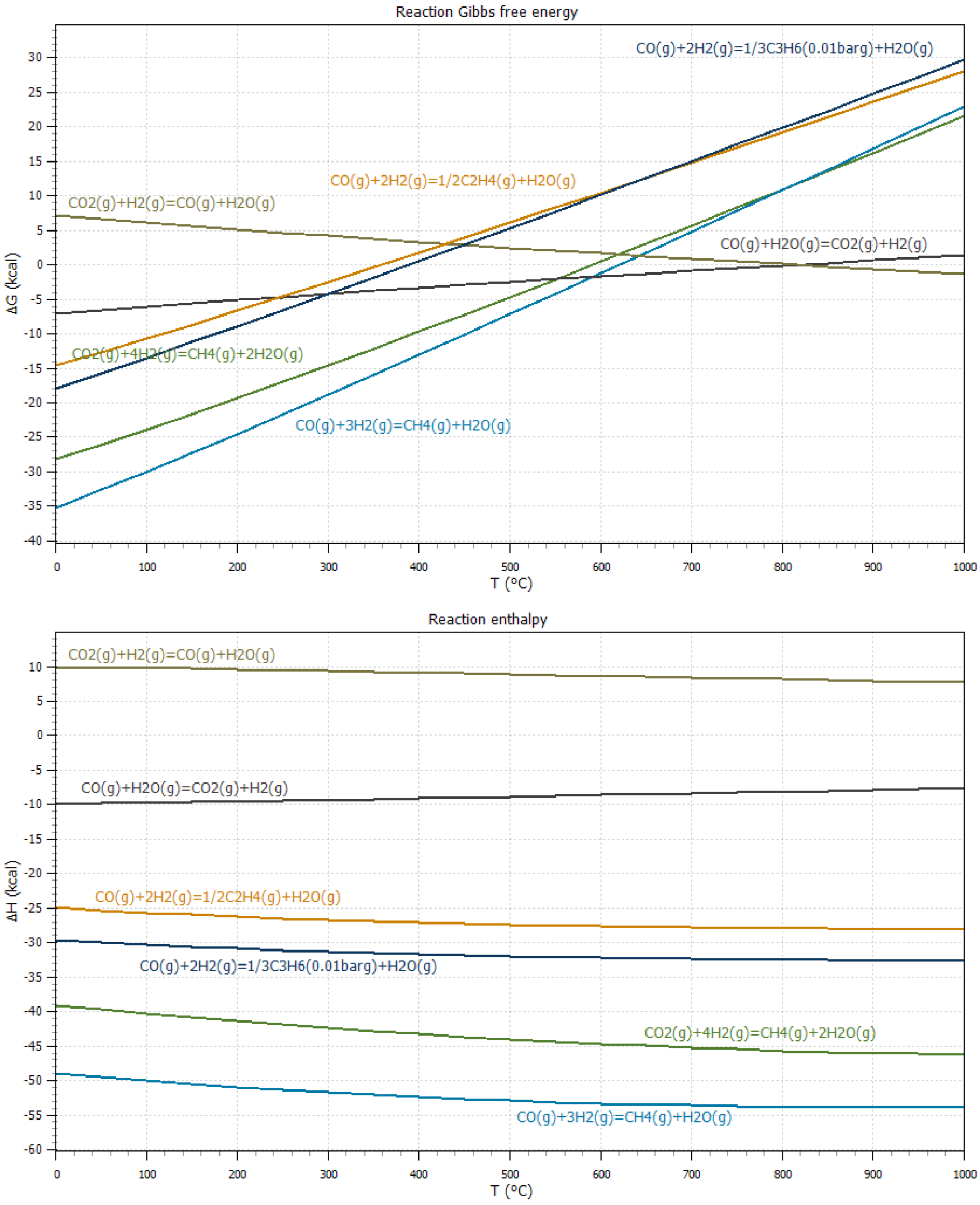

Figure S6. Thermodynamic calculations for the possible reactions taking place during CO/CO_2 hydrogenation using the CSR sample. The top panel displays the Gibbs free energy and the bottom panel the reaction enthalpy.

2.3. Catalytic Performance of Coarse Solid Residue (CSR) and the Fe/SiO_2 Catalyst Material in Biomass-derived Syngas Conversion

Table S1. Catalytic performance of coarse solid residue (CSR) at various temperatures, *P*=5 bar, $CO:CO_2:H_2:N_2$=4.5:2.5:3:1, and gas hourly space velocity (GHSV)=3400 h^{-1}. Per temperature step, 12 h of catalytic testing was performed with 1 gas chromatography (GC) injection per 23 min.

Temperature (°C)	Total C conversion (%)	Methane (%)	C_2 (%)	C_2 O/P[a]	C_2 olefin yield (%)	C_3 (%)	C_3 O/P[a]	C_3 olefin yield (%)
250	10.9 ± 0.5	79.6 ± 10.4	19.8 ± 8.7	1.18 ± 0.26	2.55			
350	13.1 ± 0.6	66.9 ± 11.7	26.6 ± 10.0	1.29 ± 0.24	4.48	6.14 ± 12.2	0.87 ± 0.19	0.70
450	16.0 ± 0.7	51.5 ± 6.9	33.4 ± 10.2	1.57 ± 0.29	8.37	14.2 ± 6.7	0.82 ± 0.13	1.87

[a] O/P stands for olefin/paraffin ratio

Table S2. Average catalytic performance of coarse solid residue (CSR) over 24 h time-on-stream at *T*=450°C, *P*=5 bar, $CO:CO_2:H_2:N_2$=4.5:2.5:3:1, and gas hourly space velocity (GHSV)=3400 h^{-1}. A gas chromatography (GC) injection was performed every 23 min.

Total C conversion (%)	Methane (%)	C_2 (%)	C_2 O/P[a]	C_3 (%)	C_3 O/P[a]	C_4 (%)	C_4 O/P[a]
14.7 ± 5.9	59.3 ± 4.5	28.6 ± 3.5	1.15 ± 0.42	11.6 ± 3.4	0.80 ± 0.23	0.48 ± 0.94	3.87 ± 1.56

[a] O/P stands for olefin/paraffin ratio

Table S3. Catalytic performance of Fe/SiO_2 at various temperatures, *P*=5 bar, $CO:CO_2:H_2:N_2$=4.5:2.5:3:1, and gas hourly space velocity (GHSV)=3400 h^{-1}. Per temperature step, 12 h of catalytic testing was performed with 1 gas chromatography (GC) injection per 23 min.

Temperature (°C)	Total C conversion (%)	Methane (%)	C_2 (%)	C_2 O/P[a]	C_3 (%)	C_3 O/P[a]	C_4 (%)	C_4 O/P[a]
250	17.0 ± 0.2	64.6 ± 7.8	31.0 ± 7.6	1.18 ± 0.56	1.48 ± 0.68	1.87 ± 0.27		
350	21.7 ± 0.1	56.8 ± 9.0	24.6 ± 4.3	1.05 0.10±	16.4 ± 4.4	1.7 0.458 ±	2.20 ± 4.12	2.63 ± 1.20
450	24.9 ± 1.0	36.9 ± 2.4	37.4 ± 5.9	0.87 ± 0.29	20.9 ± 1.2	1.43 ± 0.14	4.77 ± 0.95	0.70 ± 0.08

[a] O/P stands for olefin/paraffin ratio

Table S4. Average catalytic performance of Fe/SiO_2 over 20 h time-on-stream at *T*=450°C, *P*=5 bar, $CO:CO_2:H_2:N_2$=4.5:2.5:3:1, and gas hourly space velocity (GHSV)=3400 h^{-1}. A gas chromatography (GC) injection was performed every 23 min.

Total C conversion (%)	Methane (%)	C_2 (%)	C_2 O/P[a]	C_3 (%)	C_3 O/P[a]	C_4 (%)	C_4 O/P[a]

16.2 ± 0.2	53.3 ± 3.0	28.2 ± 2.0	0.87 ± 0.49	16.4 ± 1.6	1.37 ± 0.36	2.09 ± 2.20	1.15 ± 0.68

[a] O/P stands for olefin/paraffin ratio

2.4. X-ray Diffraction of the K-Fe/SiO_2 Sample

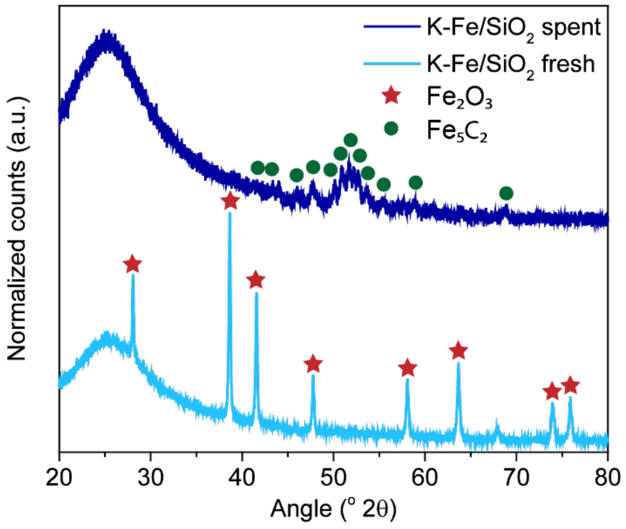

Figure S7. X-ray diffraction (XRD) patterns of the K-Fe/SiO_2 (0.71 wt.% K and 7.6 wt.% Fe) sample after calcination (fresh) containing Fe_2O_3 and after consecutive CO_2 hydrogenation, Fischer-Tropsch-to-Olefins (FTO), and CO_2 hydrogenation containing the Hägg carbide (Fe_5C_2).

2.5. Catalytic Performance for the CO_2 Hydrogenation and the Fischer-Tropsch-to-Olefins Process

Table S5. Average catalytic performance in (consecutively) the CO_2 hydrogenation reaction, the Fischer-Tropsch to-Olefins (FTO) reaction, and the CO_2 hydrogenation reaction of the coarse solid residue (CSR) sample, Fe/SiO_2 catalyst, and the K-Fe/SiO_2 catalyst. The CO_2 hydrogenation steps were carried out at T=250°C, P=5 bar, H_2/CO_2=3, and gas hourly space velocity (GHSV)=3070 h^{-1}, while the FTO step was carried out at T=350°C, P=5 bar, CO/H_2=1.5, and GHSV=2425 h^{-1}. The hydrocarbon selectivities displayed are CO and/or CO_2-free. Prior to the first CO_2 hydrogenation step, the samples were pre-reduced at 450°C in N_2/H_2=2 for 1 h. The complete set of measurement points (recorded with gas chromatography (GC) with 1 injection per 23 min) can be found in **Figure 4** in the main text.

**Reaction**	**Catalyst**	**Conversion (%)**	**CO or CO_2 (%)**	**CH_4 (%)**	**C_2 (%)**	**C_3 (%)**	**C_4 (%)**	**C_5 (%)**
CO_2 hydrogenation	CSR	1.91	91.9	79.4	20.6			
	Fe/SiO_2	2.13	0	87.3	7.59	4.92	0.24	
	K-Fe/SiO_2	2.04	30.4	71.9	17.1	10.4	1.48	
FTO	CSR	2.11	73.1	39.6	36.0	20.9	3.56	
	Fe/SiO_2	2.69	14.6	66.7	14.0	12.7	6.69	1.15
	K-Fe/SiO_2	2.49	27.5	64.3	11.0	14.8	9.85	4.09

CO_2 hydrogenation	CSR	1.90	99.4	92.7	7.29	
	Fe/SiO_2	2.26	48.0	92.2	7.12	0.71
	$K\text{-}Fe/SiO_2$	1.89	58.1	89.9	9.39	0.73

Table S6. Average olefin/paraffin (O/P) ratios during (consecutively) the CO_2 hydrogenation reaction, the Fischer-Tropsch-to-Olefins (FTO) reaction, and the CO_2 hydrogenation reaction of the coarse solid residue (CSR) sample, the Fe/SiO_2 sample, and the K-Fe/SiO_2 sample. The CO_2 hydrogenation steps were carried out at T=250°C, P=5 bar, H_2/CO_2=3, and gas hourly space velocity (GHSV)=3070 h^{-1}, while the FTO step was carried out at T=350°C, P=5 bar, CO/H_2=1.5, and GHSV=2425 h^{-1}. Prior to the first CO_2 hydrogenation step, the samples were pre-reduced at 450°C in N_2/H_2=2 for 1 h. The complete set of measurement points (recorded with gas chromatography (GC) with 1 injection per 23 min) can be found in **Figure 4** in the main text.

**Reaction**	**Catalyst**	**O/P C_2[a]**	**O/P C_3[a]**	**O/P C_4[a]**	**O/P C_5[a]**
CO_2 hydrogenation	CSR	2.07 ± 0.44			
	Fe/SiO_2	2.09 ± 0.43	2.31 ± 0.58	2.63 ± 1.10	
	$K\text{-}Fe/SiO_2$	3.98 ± 0.50	2.52 ± 0.30	4.68 ± 0.86	
FTO	CSR	10.8 ± 1.4	15.3 ± 2.6	25.9 ± 7.7	
	Fe/SiO_2	13.0 ± 3.7	13.9 ± 3.3	20.7 ± 5.9	6.67 ± 4.59
	$K\text{-}Fe/SiO_2$	18.8 ± 4.0	18.0 ± 3.3	36.2 ± 6.8	8.50 ± 3.90
CO_2 hydrogenation	CSR	1.40 ± 0.45			
	Fe/SiO_2	1.72 ± 0.54	0.71 ± 0.38		
	$K\text{-}Fe/SiO_2$	3.23 ± 2.04	1.23 ± 0.40		

[a] O/P stands for olefin/paraffin ratio

2.6. The Coarse Solid Residue with Fe Carbides Twice as Active as Metallic Fe for CO_2 Hydrogenation

The catalytic performance of the CSR sample as well as the Fe/SiO_2 catalyst for CO_2 hydrogenation and Fischer-Tropsch-to-Olefins (FTO) were evaluated at 450°C. Prior to the reaction, the samples were pre-treated at 450°C in N_2/H_2=2 for 1 h to transform Fe_2O_3, as present in the fresh catalysts, in Fe/Fe_3O_4. Then, the CO_2 hydrogenation performance was tested for 24 h at T=450°C, P=5 bar, and H_2/CO_2=3 (**Table S7**). Consecutively, Fe carbide (Hägg, Fe_5C_2) was formed during a 24 h step under the FTO conditions T=450°C, P=5 bar, and H_2/CO=0.5. Finally, a second CO_2 hydrogenation step of 24 h was performed using the same reaction conditions as in the first CO_2 hydrogenation step. When comparing the performance of Fe_5C_2 present in CSR after the FTO step to Fe/Fe_3O_4 present in CSR after the pre-reduction step (**Table S7**), it appeared that the Fe carbide phase converted about twice as much CO_2 as the Fe/Fe_3O_4 phase. The C_{2+} selectivity was slightly higher for the Fe/Fe_3O_4 phase compared to the Fe_5C_2 phase. Both the iron carbide phase and the Fe/Fe_3O_4 phase were thus active phases for the conversion of CO_2. Though, to obtain the highest C_{2+} yield with CSR, it would be more efficient to perform CO_2 hydrogenation with the Fe/Fe_3O_4 phase. Also noteworthy is that CSR displayed a high selectivity towards CO during the CO_2 hydrogenation reaction; 74.5 and 80.4% for the Fe/Fe_3O_4 and Fe_5C_2 phase, respectively. Producing CO from CO_2 could be an interesting strategy for two-step CO_2 valorization processes. In the first step, CO_2 would be converted to the more reactive building block CO. In the second step, CO would be converted into valuable long-chain hydrocarbons or oxygenates. Besides, co-feeding CO and CO_2 seemed an effective strategy to achieve a higher C_{2+} selectivity (**Figure 3** in the main text).

Table S7. Consecutive CO_2 hydrogenation, Fischer-Tropsch-to-Olefins (FTO) and CO_2 hydrogenation experiments with coarse solid residue (CSR) and Fe/SiO_2. The samples contained either Fe/Fe_3O_4 in the first CO_2 hydrogenation step (pre-treated at 450°C in N_2/H_2=2 for 1 h) or Fe carbide in the second CO_2 hydrogenation step (T=450°C, P=5 bar, H_2/CO_2=3, gas hourly space velocity (GHSV)=3070 h^{-1}, 24 h time-on-stream). The Fe carbide (Hägg, Fe_5C_2) was formed under FTO conditions (T=450°C, P=5 bar, H_2/CO=0.5, GHSV=3400 h^{-1}, 24 h time-on-stream) in between the two CO_2 hydrogenation steps. The iron phase in the FTO step is indicated as "mix", since the transformation of Fe/Fe_3O_4 occurs during the FTO step.

Catalyst	Testing conditions	Fe phases	Conversion (%)	CO selectivity (%)	CO_2 selectivity (%)	CH_4 selectivity (%)	C_{2+} selectivity (%)
CSR	CO_2	Fe/Fe_3O_4	8.40	74.5	n.a.	86.5	13.5
	FTO	mix	39.6	n.a.	13.0	59.3	40.7
	CO_2	Fe_5C_2	16.0	80.4	n.a.	95.2	4.81
Fe/SiO_2	CO_2	Fe/Fe_3O_4	10.3	0.0	n.a.	93.8	4.12
	FTO	mix	45.2	n.a.	17.2	72.9	27.0
	CO_2	Fe_5C_2	8.48	60.4	n.a.	95.9	6.18

[a] The hydrocarbon selectivities reported are CO and/or CO_2-free.

2.7. *Operando* X-ray Diffraction with the Coarse Solid Residue Sample

Table S8. Catalytic performance as measured with gas chromatography (GC), with one injection per 23 min, during the *operando* X-ray diffraction (XRD) experiment with coarse solid residue (CSR). The sample was pre-treated in pure H_2 for 1 h at 450°C prior to the reaction. Fischer-Tropsch-to-Olefins (FTO) reaction conditions were T=450°C, P=5 bar, and CO:H_2:He=2.25:1.5:1 for a total of 70 h time-on-stream (TOS). The averages of 0-10 h TOS are compared to the averages of 60-70 h TOS.

Product	Conversion or selectivity at 0-10 h (%)	Conversion or selectivity at 60-70 h (%)
CO conversion	25.9 ± 0.3	33.9 ± 0.3
Methane	87.9 ± 2.27	75.8 ± 1.2
Ethane	2.94 ± 0.81	4.62 ± 0.53
Ethene	0.79 ± 0.13	2.01 ± 0.09
Propane	0.80 ± 0.20	0.93 ± 0.04
Propene	0.73 ± 0.31	2.08 ± 0.08
iso-Butane	0	0
n-Butane	0.02 ± 0.04	0.15 ± 0.06
Acetylene	0	0
trans-2-butene	0	0.03 ± 0.04
1-butene	0	0.18 ± 0.13
iso-butene	0	0.04 ± 0.05
cis-2-butene	0	0.01 ± 0.02
isopentane / 2-methyl-butane	0	0
pentane	0	0.06 ± 0.05
1,3-butadiene	0	0
neopentane / 2,2-dimethylpropane	0	0
3-methyl-1-butene	0	0
trans-2-pentene	0	0

2-methyl-2-butene	0	0.01 ± 0.03
1-pentene	0	0
2-methyl-1-butene	0	0

2.8. Catalytic Performance of the Coarse Solid Residue (CSR) Sample in Biomass-derived Syngas Conversion at *P*=20 bar

Table S9. Average olefin/paraffin (O/P) ratio in the hydrocarbon products made by coarse solid residue (CSR) at varying temperatures (6 h per temperature), *P*=20 bar, $CO:CO_2:H_2:N_2$=4.5:2.5:3:1, and gas hourly space velocity (GHSV)=3400 h^{-1}. A gas chromatography (GC) injection was performed every 23 min.

Temperature (°C)	O/P C_2[a]	O/P C_3[a]	O/P C_4[a]
250	1.75 ± 1.75	2.34 ± 2.28	
300	2.61 ± 2.13	2.44 ± 2.24	1.65 ± 0.17
350	6.52 ± 3.89	3.43 ± 3.63	1.61 ± 0.93
400	8.72 ± 6.82	5.06 ± 2.34	2.44 ± 1.22
450	13.3 ± 2.7	14.0 ± 3.4	7.41 ± 3.64

[a] O/P stands for olefin/paraffin ratio
